# Dual-Temperature/pH-Sensitive Hydrogels with Excellent Strength and Toughness Crosslinked Using Three Crosslinking Methods

**DOI:** 10.3390/gels10070480

**Published:** 2024-07-19

**Authors:** Jiaqi Wang, Wanying Yang, Yutong Li, Xuerong Ma, Yuxin Xie, Guangyan Zhou, Shouxin Liu

**Affiliations:** Key Laboratory of Applied Surface and Colloid Chemistry, Ministry of Education, School of Chemistry and Chemical Engineering, Shaanxi Normal University, Xi’an 710119, China; wjqsxdtdx@163.com (J.W.); yangwy15935903470@163.com (W.Y.); lyt15045546662@163.com (Y.L.); 18392883393@163.com (X.M.); 15026099832@163.com (Y.X.); 19192540226@163.com (G.Z.)

**Keywords:** hydrogel, triple crosslinking, stereocomplexation, temperature/pH dual response, free-radical copolymerization

## Abstract

Hydrogels are widely used as excellent drug carriers in the field of biomedicine. However, their application in medicine is limited by their poor mechanical properties and softness. To improve the mechanical properties of hydrogels, a novel triple-network amphiphilic hydrogel with three overlapping crosslinking methods using a one-pot free-radical polymerization was synthesized in this study. Temperature-sensitive and pH-sensitive monomers were incorporated into the hydrogel to confer stimulus responsiveness, making the hydrogel stimuli-responsive. The successful synthesis of the hydrogel was confirmed using techniques, such as proton nuclear magnetic resonance spectroscopy (^1^H NMR), Fourier-transform infrared spectroscopy (FT-IR), and X-ray diffraction (XRD). In order to compare and analyze the properties of physically crosslinked hydrogels, physically–chemically double-crosslinked hydrogels, and physically–chemically clicked triple-crosslinked hydrogels, various tests were conducted on the gels’ morphology, swelling behavior, thermal stability, mechanical properties, and drug loading capacity. The results indicate that the triple-crosslinked hydrogel maintains low swelling, high mechanical strength, and good thermal stability while not significantly compromising its drug delivery capability.

## 1. Introduction

Nowadays, cancer is a difficult problem plaguing people [1,2,3,4,5]. How to carry out targeted therapy for cancer is one of the top issues researchers are concerned about, and targeted therapy needs carriers [6,7,8,9,10,11]. Because hydrogels have the functions of storing drugs, controlling the speed of drug release, good biocompatibility, and flexibility, similar to that of tissue [12,13,14], they have a wide range of applications in the field of biomedicine.

Smart-responsive hydrogels (also known as stimuli-responsive hydrogels) are a class of hydrogels that can change their properties in response to environmental stimuli; when subjected to physical or chemical stimuli, the hydrogels will swell or shrink [15,16,17,18,19]. The types of stimuli that elicit responses are mainly classified into the following categories: temperature [20,21,22], pH [23,24], redox [25,26], light [27,28], electricity [29,30], magnetism [31,32], chemicals [33,34], etc. There are both single-stimuli-responsive hydrogels and multi-stimuli-responsive hydrogels [35,36]. In terms of hydrogel’s drug-carrying function, the drug delivery can be controlled by the release mechanisms of diffusion and dissolution [37,38] through the stimulatory response of hydrogels to the external environment. In addition, temperature and pH are two important physiological parameters of the human body; in human tissue lesions, these two values often change [39,40,41,42], so the selection of hydrogels with appropriate temperature and pH stimulation responses as carriers for targeted therapy is necessary. Hydrogels loaded with drugs in their focal site can be used for the persistent release of drugs in the reservoir, which not only realizes the effective controlled release of drugs but also improves the utilization rate of drugs. Thus, the drug-carrying function of hydrogels has a wide range of applications in biomedical fields [43,44,45,46,47,48,49].

Since hydrogels are three-dimensional crosslinked polymer materials with high water contents [50,51,52,53], their mechanical properties are an important indicator for their use as a biomedical material [54,55,56,57]. However, in water or under complex physiological conditions, hydrogels tend to absorb and swell due to the difference in osmotic pressure, which drastically reduces their mechanical properties [58], greatly limiting the practical application of hydrogels in the biomedical field. How to prepare hydrogels with anti-swelling properties has become a hot spot for researchers [59,60,61,62,63]. For example, injectable hydrogel adhesives that can rapidly adhere to wet tissues and resist swelling are urgently needed for fast hemostasis and wound healing in surgical applications. However, hydrophilic adhesives often exhibit swelling behavior, leading to significant volume expansion. This swelling can weaken cohesive forces, impair adhesion strength, and result in poor performance and unpredictable outcomes, including wound reopening and bleeding. Therefore, there is a critical need to develop injectable hydrogel adhesives that integrate rapid adhesion and anti-swelling properties for achieving fast hemostasis and wound sealing in surgical applications. Furthermore, the skeletal structure of biomaterials plays a crucial role in tissue engineering, providing mechanical support and developmental guidance for cell growth and the formation of new tissue. The superior viscoelasticity, anti-swelling properties, and ECM-like characteristics of hydrogels allow them to be used as cell support scaffolds for soft tissue regeneration.

In the hydrogels we have studied before [64,65], there are two crosslinking methods: physical crosslinking hydrogels and physical–chemical double-crosslinking hydrogels [66,67]. The structure of a purely physically crosslinked hydrogel is unstable and easily disrupted by external forces. The stability of a physically–chemically double-crosslinked hydrogel is improved to some extent, but even after complete swelling, the minimum swelling ratio is still 17%. It is preferable for hydrogels used to deliver drugs in human tissues to have minimal volume swelling, as this minimizes their impact on human tissues. Therefore, in this work, three hydrogels with dual responses to temperature and pH were prepared based on three different crosslinking methods, using biocompatible polylactic acid as a raw material. The aim was to reduce the swelling ratio of the hydrogels and improve their mechanical properties by changing the crosslinking method. The monomers used, such as polylactic acid, thermosensitive monomers 2-methyl-2-acylate-2-(2-methoxyethoxy) ethyl ester (MEO_2_MA) and oligoethylene glycol methyl ether methacrylate (OEGMA), pH-sensitive monomer *N,N*-Diethylaminoethyl methacrylate (DEAEMA), and click chemistry bonds, all exhibit good biocompatibility, which is essential for drug delivery applications.

## 2. Results and Discussion

### 2.1. Synthesis

**Scheme 1:**
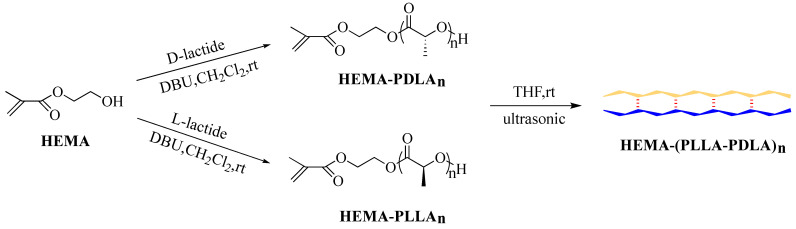
Synthesis of stereocomplexes HEMA-(PLLA-PDLA)_n_.

**Scheme 2:**
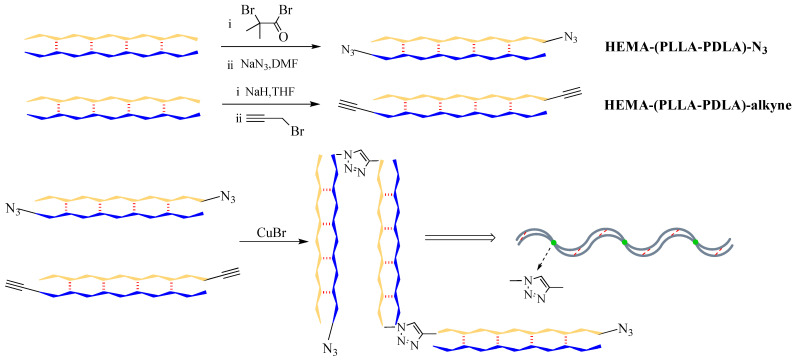
Synthesis of HEMA-(PLLA-PDLA)-N_3_, HEMA-(PLLA-PDLA)-alkyne and triazole polymers.

**Scheme 3:**
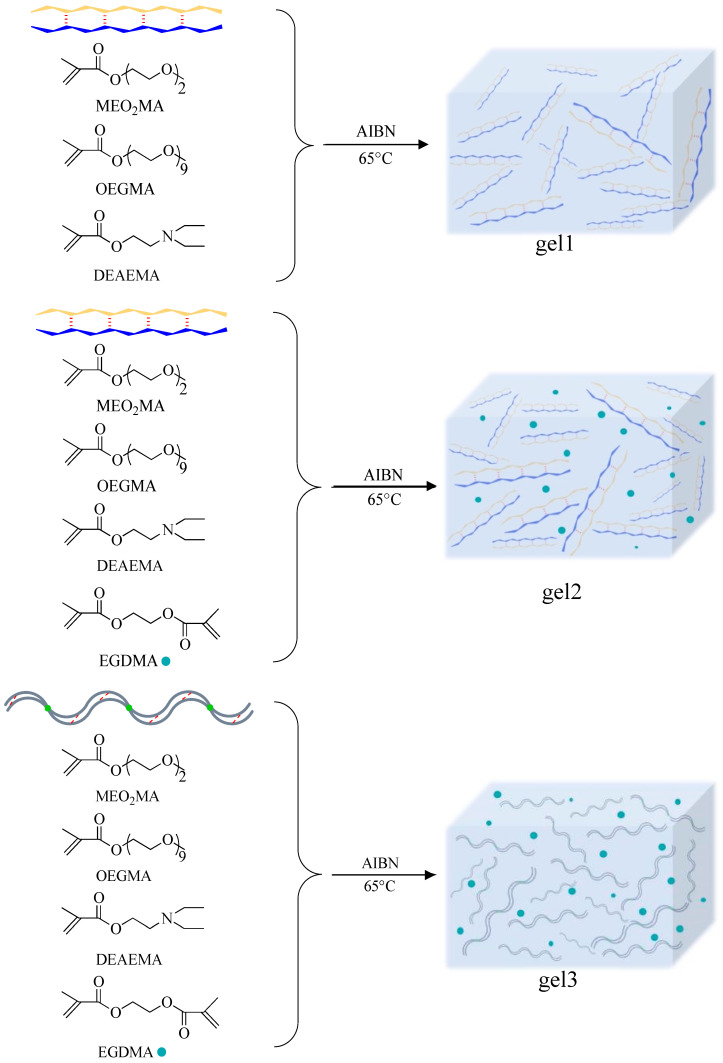
Synthesis of hydrogels.

Hydroxyethyl methacrylate (HEMA) and lactide were synthesized by ring-opening polymerization catalyzed by DBU to synthesize HEMA-PLLA_n_ and HEMA-PDLA_n_ [64], which were ultrasonicated to form the stereocomplexes, HEMA-(PLLA-PDLA)_n_, which were crosslinked together via intermolecular hydrogen bonding. The synthesis steps are shown in Scheme 1. The stereocomplexes were then azidized and alkynylated to synthesize HEMA-(PLLA-PDLA)-N_3_ and HEMA-(PLLA-PDLA)-alkyne, respectively, and the azide and alkynylate were subjected to a click reaction [68] to generate triazole in a certain ratio. They were then catalyzed by CuBr to form click crosslinks. The synthesis steps are shown in Scheme 2. The synthesis of hydrogels was carried out next, all of which were synthesized by one-pot free radical copolymerization. HEMA-(PLLA-PDLA)_n_, thermosensitive monomers MEO_2_MA and OEGMA, and pH-sensitive monomer DEAEMA synthesized physically crosslinked hydrogels. HEMA-(PLLA-PDLA)_n_, thermosensitive monomers MEO_2_MA, OEGMA, pH-sensitive monomer DEAEMA, and chemical crosslinker EGDMA synthesized physically–chemically double-crosslinked hydrogel. Triazole polymer, thermosensitive monomers MEO_2_MA and OEGMA, pH-sensitive monomer DEAEMA, and chemical crosslinker EGDMA synthesized physically–chemically clicked triple-crosslinked hydrogel, and the synthesis procedure is described in Scheme 3. In Scheme 3, all three gels have a three-dimensional mesh structure, just to highlight that the crosslinking mode is not drawn. Physical crosslinking points were drawn in gel1, physical crosslinking points with chemical crosslinking points in gel2, physical crosslinking points, chemical crosslinking points, and clicking chemical crosslinking points in gel3, and each crosslinking point interacts with the others, which together form the internal structure of the hydrogel.

Three different gels were synthesized by three different crosslinking methods, and the properties of the gels were tested to compare and analyze the effects of different crosslinking methods on the gel properties. The synthesized data of each gel are shown in Table 1.

### 2.2. Structural Characterization

#### 2.2.1. Structural Characterization of HEMA-PLLA_n_, HEMA-PDLA_n_ and HEMA-(PLLA-PDLA)

**Figure 1:**
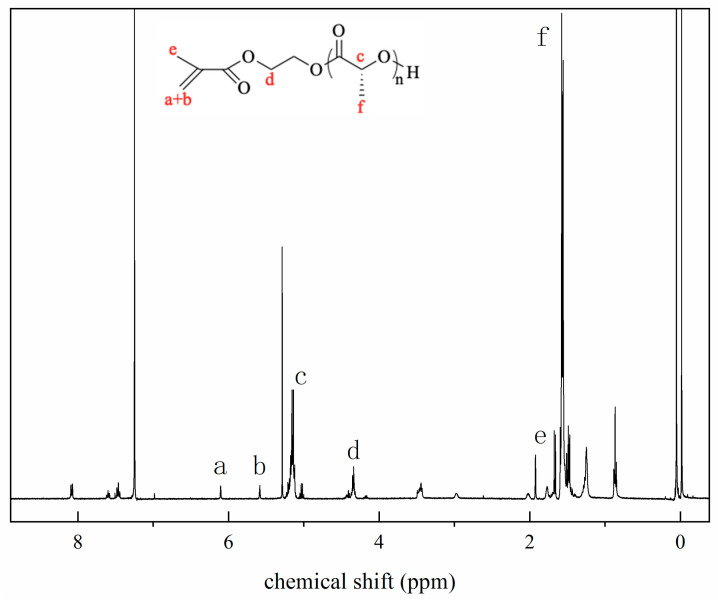
^1^H NMR diagram of HEMA-PLLA_30_.

The structural characterization and successful synthesis of the macromonomers HEMA-PLLA and HEMA-PDLA were demonstrated based on ^1^H NMR and FT-IR spectra. In the present work, the macromonomers used were all polymerized with a degree of polymerization of 30, and the ^1^H NMR spectra of macromonomers polymerized with a degree of polymerization of 30 are also shown in Figure 1. In the figure, 6.10 ppm (a) and 5.58 ppm (b) are proton peaks of the double bond, 5.27–5.08 ppm (c) with 1.63–1.53 ppm (f) is hypromethyl and methyl proton peaks of the PLA repeating unit, 4.41–4.28 ppm (d) shows proton peaks of the methylene of the O=COCH_2_CH_2_O- moiety, 1.92 ppm (e) is the methyl proton peak of the -(CH_3_)C=CH_2_ group; the figure indicates the successful synthesis of HEMA-PLLA_30_. The ^1^H NMR diagrams of HEMA-PLLA_30_ and HEMA-PDLA_30_ are consistent and will not be repeated.

The number of LA units *n* in HEMA-PLLA_n_ and HEMA-PDLA_n_ is calculated using the following formula:

$$\;n=\frac{A_{c}}{A_{a}}$$

where *A_c_* and *A_a_* represent the integral area of c and a, respectively. In Figure 1, for HEMA-PLLA_30_, *A_a_* = 1.00 and *A_c_* = 29.72, so *n* = 29.72 ≈ 30.

**Figure 2:**
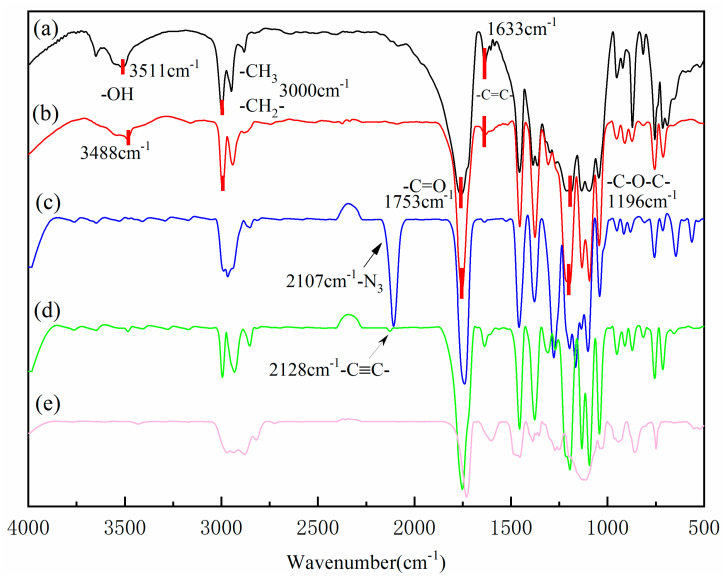
FT−IR spectra of the HEMA-PLLA_30_ (**a**), HEMA-(PLLA-PDLA) (**b**), HEMA-(PLLA-PDLA)-N_3_ (**c**), HEMA-(PLLA-PDLA)-alkyne (**d**), and gel3 (**e**).

Figure 2a,b show the FT-IR spectra of the macromonomers HEMA-PLLA_30_ and HEMA-(PLLA-PDLA). In the figure, the peaks appearing at 1753 cm^−1^ and 3511 cm^−1^ (3488 cm^−1^) are the stretching vibration peaks of C=O and -OH, respectively, the peak at 1196 cm^−1^ is the stretching vibration peak of C-O-C, the peak at 1633 cm^−1^ is the stretching vibration peak of C=C, and the peak at 3000 cm^−1^ is the bending vibration and the stretching vibration of the C-H of methyl and methylene, which proves the successful synthesis of the macromonomers HEMA-PLLA_30_ and HEMA-(PLLA-PDLA). The FT-IR diagrams of HEMA-PLLA_30_ and HEMA-PDLA_30_ are consistent and will not be repeated.

**Figure 3:**
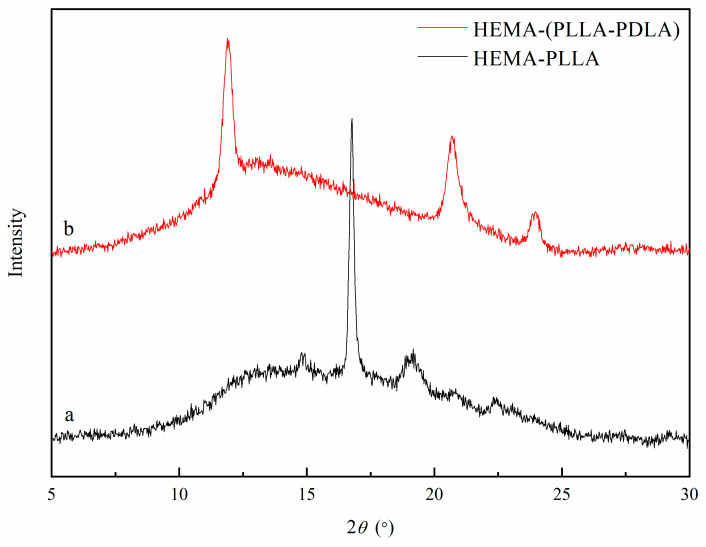
(**a**) XRD pattern of the HEMA-PLLA; (**b**) XRD pattern of the HEMA-(PLLA-PDLA).

In order to further prove that the stereocomplex HEMA-(PLLA-PDLA) is connected by hydrogen bonding between PLLA and PDLA, we carried out X-ray diffraction studies on the macromonomers HEMA-PLLA and the stereocomplex HEMA-(PLLA-PDLA), as shown in Figure 3a,b, whre the XRD curves of the HEMA-PLLA and the XRD curves of the HEMA-(PLLA-PDLA) can be found, respectively. In the (a) curve, we can see that the diffraction peaks appear at 2θ = 17°, which is the isomorphous peak of macromonomers; in the (b) curve, diffraction peaks appear at 2θ = 12°, 21°, and 24°, which is the stereo-structural feature of PLA [69]. Further, the isomorphous peak of macromonomers disappeared, which proves that the steric complexes are formed by the steric complexation of PLLA and PDLA. The successful synthesis of the stereocomplex HEMA-(PLLA-PDLA) was further demonstrated.

#### 2.2.2. Structural Characterization of HEMA-(PLLA-PDLA)-N_3_ and HEMA-(PLLA-PDLA)-Alkyne

The FT-IR spectra of HEMA-(PLLA-PDLA), HEMA-(PLLA-PDLA)-N_3_, and HEMA-(PLLA-PDLA)-alkyne are shown in Figure 2b–d. Comparing (c) with (b) and (c) shows a telescopic vibrational absorption peak of the azide group at 2107 cm^−1^, which proves that the azide treatment of the steric complex HEMA-(PLLA-PDLA)-alkyne was successful. Comparing (d) with (b) and (d) shows a stretching vibrational absorption peak of the alkyne group at 2128 cm^−1^, which proves the successful alkyneization of the stereocomplex HEMA-(PLLA-PDLA).

#### 2.2.3. Structural Characterization of Gels

The structural characterization of the gels with successful synthesis was proved based on FT-IR and XRD spectra. Figure 2c–e show the FT-IR spectra of HEMA-(PLLA-PDLA)-N_3_, HEMA-(PLLA-PDLA)-alkyne, and gel3. Comparing (c) (d) with (e), it can be seen that the azide peak and alkyne peak in (e) disappear, proving that there is a click chemical bond generated by the reaction of azide group and alkyne group in gel3.

**Figure 4:**
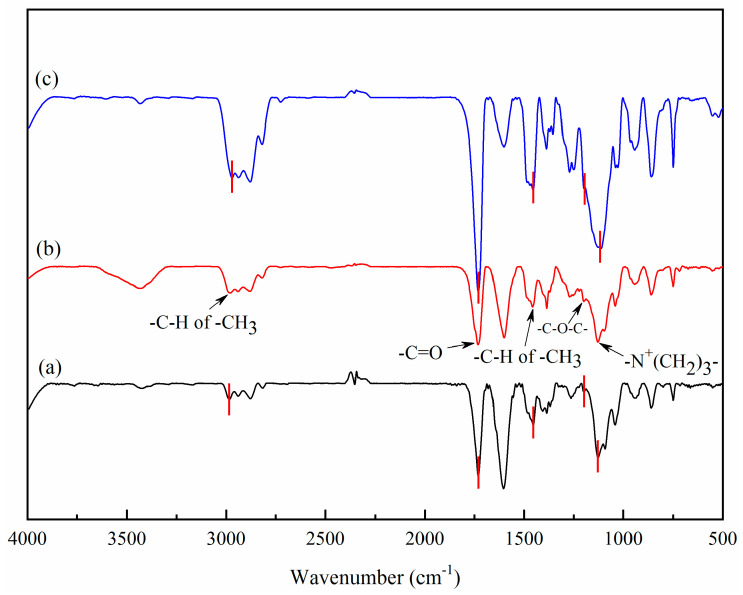
FT−IR spectra of gel1 (**a**); gel2 (**b**); gel3 (**c**).

Figure 4 shows the FT-IR spectra of the three gels. In the figure, it can be seen that all three gels showed telescopic vibration peaks of -CH_3_- with bending vibration peaks at 2988 cm^−1^ and 1458 cm^−1^; telescopic vibration peaks of -C=O- at 1729 cm^−1^; telescopic vibration peaks of -C-O-C- at 1194 cm^−1^; and vibrational absorption peaks of -N^+^(CH_2_)_3_- at 1128 cm^−1^. This proved the successful synthesis of the gels [64].

**Figure 5:**
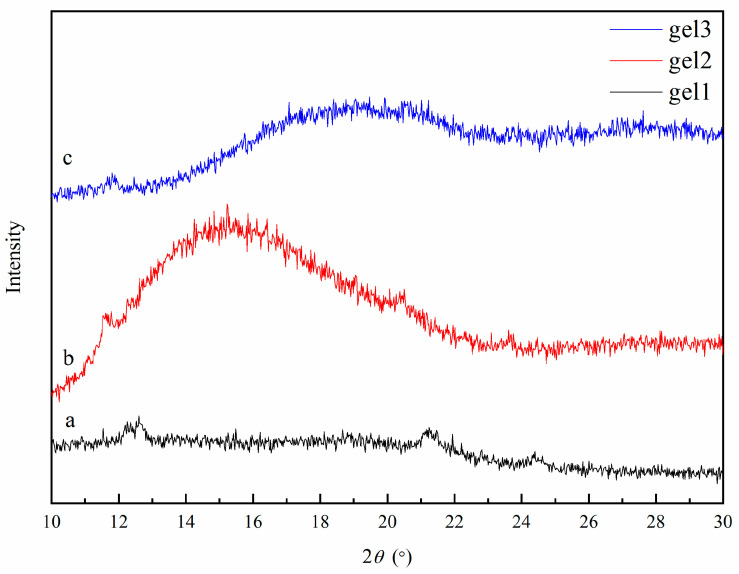
(**a**) XRD pattern of gel1; (**b**) XRD pattern of gel2; (**c**) XRD pattern of gel3.

The physical crosslinking states of the three gels can be determined by X-ray diffraction. As shown in Figure 5, diffraction peaks were observed at 2θ = 12° for all three gels, with gel1 exhibiting prominent diffraction peaks at around 2θ = 21° and 24°. The diffraction peaks of gel2 and gel3 gradually became less pronounced, with gel3 showing the least distinct diffraction peaks. This is perhaps because of the overlapping of crosslinking modes, which means gel2 is a physico-chemical double-crosslinked hydrogel, synthesized by two crosslinking modes, and gel3 is a physico-chemical-click triple-crosslinked hydrogel, synthesized by three crosslinking modes. As the types of crosslinking modes are increased, this affects the diffraction peaks formed by the physical crosslinks in the gel. According to the literature, it is proved that the presence of diffraction peaks at 2θ = 12° can prove that steric complex crosslinking occurs in all three types of gels [70].

### 2.3. Morphology Analysis of Gels

**Figure 6:**
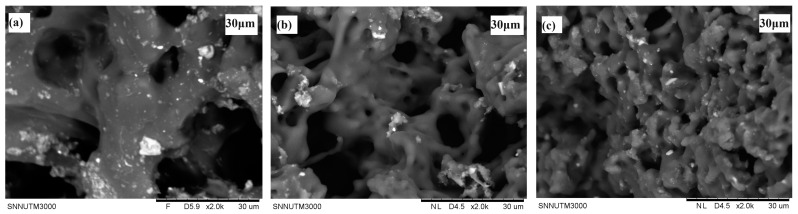
SEM of gel1 (**a**); gel2 (**b**); gel3 (**c**).

Firstly, we observed and analyzed the surface morphology of the gels, using a bench-top scanning electron microscope, to obtain Figure 6, in which (a) (b) (c) are the scanning electron microscope images of gel1, gel2, and gel3, in that order. From the figure, it can be seen that the three crosslinked gels have many pores; the pores of gel1 are the largest, followed by gel2, and the smallest is gel3. The pores of gel1, gel2, and gel3 gradually become smaller because with the increase in crosslinking mode, the crosslinking density of the gels increases, the point of action increases, and the pores gradually become smaller [71].

### 2.4. Temperature Sensitivity Test of Gels

**Figure 7:**
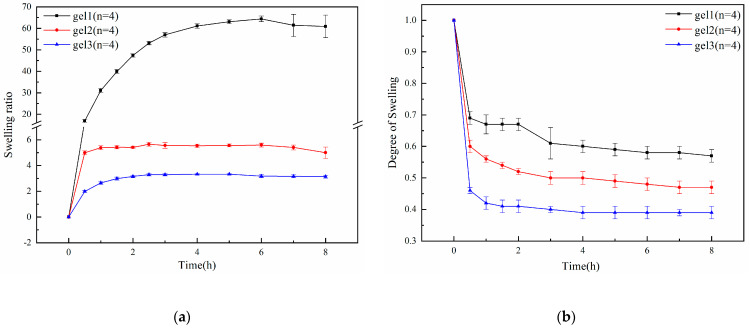
(**a**) Swelling rate of gels at 25 °C, where n is the number of samples; (**b**) deswelling rate of gels at 37 °C, where n is the number of samples.

Since the synthesized gels are temperature-responsive, in order to verify the temperature sensitivity of the gels, dynamic swelling experiments of three gels were conducted in secondary deionized water at 25 °C and pH = 7, resulting in Figure 7a. The gels that had swollen and equilibrated in the secondary deionized water at 25 °C and pH = 7 were transferred to the secondary deionized water at 40 °C and pH = 7, and the deswelling kinetics of the gels was investigated, as shown in Figure 7b. As can be seen from Figure 7a, the gels with different crosslinking modes show obvious swelling, which is because the hydrophilic part of the gel, P(MEO_2_MA-*co*-OEGMA), combines with the water molecules to form hydrogen bonds at 25 °C, causing the gel to absorb water and swell, and the hydrogels with different crosslinking modes have different swelling rates. Among them, as shown in Table 2, the physical crosslinked hydrogel has the highest swelling rate, up to more than 60, followed by physical–chemical double-crosslinked hydrogel with a swelling rate of about 5; the triple-crosslinked hydrogel has the lowest swelling rate, only about 3. This is because with the increase in crosslinking mode, the crosslinking point increases, i.e., the crosslinking point for the physical crosslinking gel, physical–chemical double-crosslinking gel, and physical–chemical click triple-crosslinking gel increases in turn, and the pore size of the gel decreases in turn, resulting in the swelling rate decreasing. The triple-crosslinked gel has a lower swelling rate, indicating that the gel does not absorb water and lead to a significant increase in volume. The larger the swelling rate, the more the gel absorbs water and swells, i.e., the larger the volume of the gel. According to Table 2, comparing the swelling rate of gel3 with that of gel1 at 8 h, the swelling rate of gel1 was 60.94 and that of gel3 was 3.14, which indicated that there was a significant increase in the volume of gel1 and an insignificant increase in the volume of gel3, making the hydrogel not too edematous when used for drug delivery, a feature that makes the hydrogel important as a drug-carrying medium for its role in human environments. From Figure 7b, it can be seen that the deswelling rate of gel1, -2, and -3 increased sequentially, which was attributed to the fact that with the increase in crosslinking points of the gels and the increase in crosslinking density, the structure of the gels was more stable, and the influence of the external environment became smaller, which was manifested in a larger deswelling rate. The synthesized swelling kinetics and deswelling kinetics curves of hydrogels proved that the gels were temperature-sensitive.

### 2.5. pH Sensitivity Test of Gels

**Figure 8:**
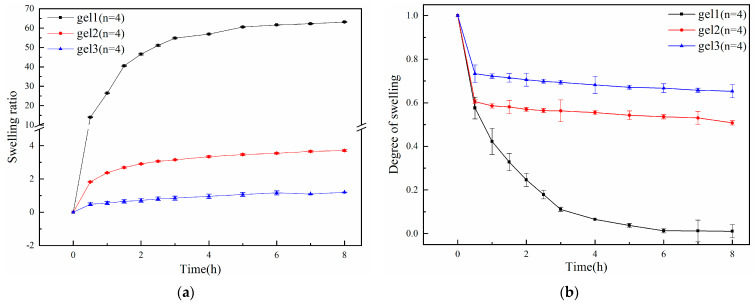
(**a**) Swelling rate of gels in solution at 37 °C and pH = 5, where n is the number of samples; (**b**) deswelling rate of gels in solution at 37 °C and pH = 9, where n is the number of samples.

Because the pH-sensitive monomer was also added to the gel, which is pH-responsive, i.e., pH-sensitive, to verify its properties, we performed swelling kinetics experiments on the gel in secondary deionized water at 25 °C, pH = 5. The hydrogel, which was completely swollen in secondary deionized water at 25 °C, pH = 5, was transferred to secondary deionized water at 25 °C, pH = 9, for the deswelling kinetics experiments which produced Figure 8a,b. As can be seen from Figure 8a and Table 3, the hydrogels with different crosslinking modes showed various degrees of swelling, and the swelling rate of the gels increased until plateauing with time. According to Table 3, comparing the dissolution rates of gel1, gel2, and gel3 at 8 h, the dissolution rates were 63.14 for gel1, 3.71 for gel2, and 1.20 for gel3, indicating that the volume increase in gel1 was significant, followed by gel2, and the volume increase in gel3 was the least significant. This is because at pH = 5, the pH value is lower than the added pH-sensitive monomer DEAEMA p*K*_a_ value of 7 and the pH-sensitive monomer protonation, resulting in electrostatic repulsion inside the gel, so the internal structure of the gel presents a stretching state [72]; the structure then becomes loose, the pores of the gel increase, and the swelling rate begins to increase. When the protonation of all the pH-sensitive monomers in the gel ends, the swelling rate of the gel does not increase, and the curve becomes flat. The gels with different crosslinking modes have different swelling rates, among which the physical crosslinking gel has the highest swelling rate at about 60, followed by the physical–chemical double-crosslinking gel at about 3, and the lowest swelling rate is the triple-crosslinking gel at only about 1. This is due to the fact that, with the increase in crosslinking modes, crosslinking points increase, the pore size of the gel decreases, and the swelling rate decreases. From Figure 8b, it can be seen that all three gels have a contraction trend with time because the environmental pH is higher than the p*K*_a_ value of DEAEMA, and deprotonation occurs from pH = 5 to pH = 9. The electrostatic effect inside the gel disappears, the structure becomes compact, the pore size of the gel becomes smaller, and the gel starts to shrink and lose water, showing the deswelling state. When the deprotonation of all pH-sensitive monomers inside the gel ends, the deprotonation rate of the gel does not decrease, and the curve becomes flat.

### 2.6. Reversibility Test of Gels

**Figure 9:**
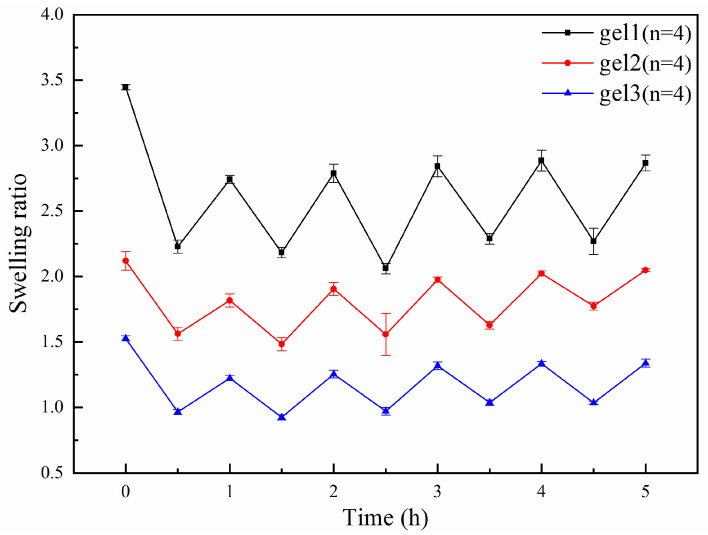
Reversible swelling of gel1, gel2 and gel3, where n is the number of samples.

Next, we put the three gels into a water bath at 23 °C and 40 °C, switched them at fixed intervals, and measured their swelling rate at each time, as shown in Figure 9, which shows that the swelling rate of the gels is basically fixed in the constant change in temperature, proving that the reversibility of the three gels was good.

### 2.7. Amphiphilicity Test of Gels

**Figure 10:**
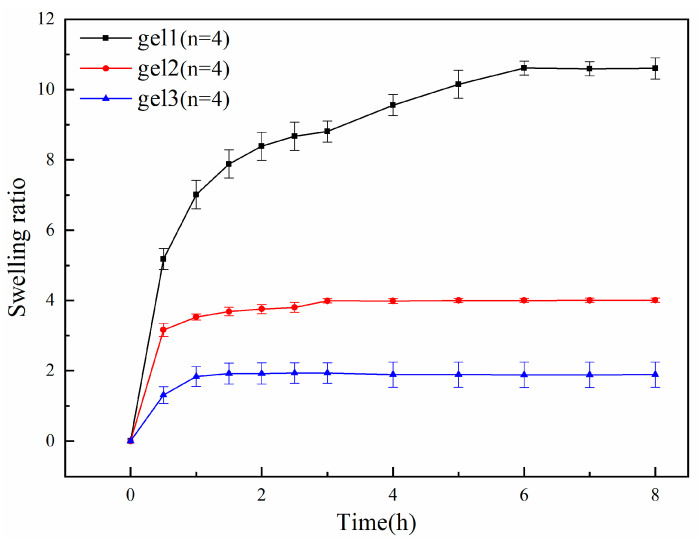
Swelling rate of gels at THF, where n is the number of samples.

Because the synthesized conetwork gels (APCNs) are a new class of crosslinked polymers [73,74] consisting of covalently bonded hydrophilic and hydrophobic polymer chains, they are both hydrophilic and hydrophobic; i.e., they are amphiphilic. In order to verify their amphiphilicity, we put the gels into the secondary deionized water and THF at 25 °C and measured the swelling rate with time, as shown in Figure 7a and Figure 10. It can be seen that the three kinds of gels were swollen in the water and THF, and the swelling rate was the first to increase rapidly and then tended to be flat, which proved that the gels had good amphiphilicity.

**Figure 11:**
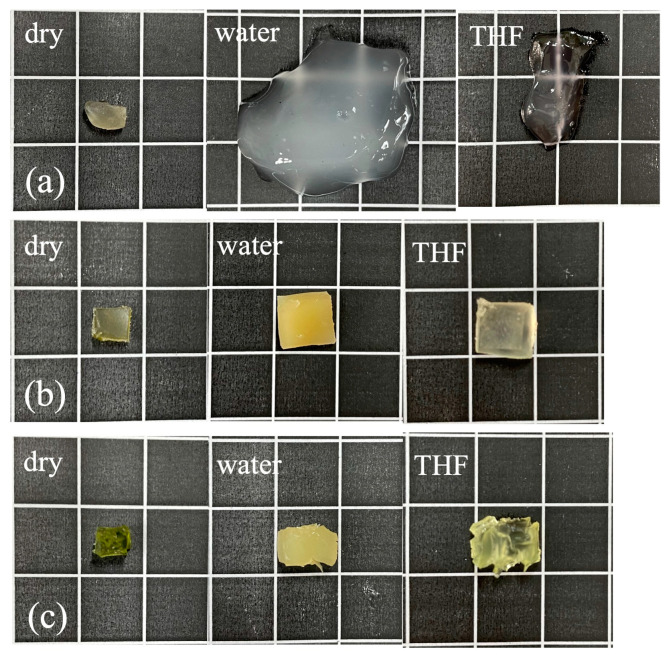
Digital photographs of gel1 (**a**), gel2 (**b**), and gel3 (**c**) in dry state, fully swollen in secondary deionized water, and fully swollen in THF.

To further prove the amphiphilicity of the gels, we photographed and recorded the dry state of the three gels, looking at the complete swelling equilibrium in water and the complete swelling equilibrium diagram in THF, respectively, with a digital camera, as shown in Figure 11. It can be seen that all of the gels underwent swelling, which further proves that all of the synthesized gels had amphiphilicity.

### 2.8. Thermal Stability Test of Gels

**Figure 12:**
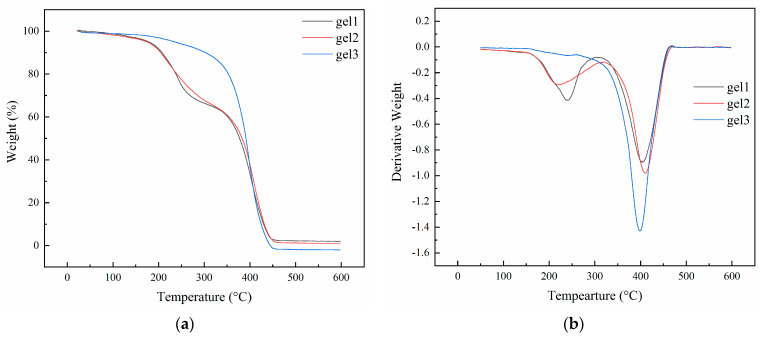
(**a**) TG diagram of gels; (**b**) DTG diagram of gels.

The thermal stability of the three gels was determined by thermogravimetric testing, which yielded Figure 12a,b. The temperature point with the largest rate of mass change on the DTG curve (thermogravimetric differential curve) corresponds to the inflection point on the TG curve (thermogravimetric curve). In the TG graph, it can be seen that gel1 and gel2 have two-times the weight loss, respectively, at about 200 °C and 340 °C as well as 200 °C and 360 °C, compared with the triple-crosslinked hydrogel only at about 350 °C, and the first loss of weight temperature is lower than the triple-crosslinked hydrogel, which indicates that the triple-crosslinked hydrogel has good thermal stability. This is due to the fact that as the crosslinking density increases, the number of action points constituting the three-dimensional network structure within the gel increases, and the relative thermal stability is gradually improved [75,76,77].

### 2.9. Mechanical Properties Tests of Gels

**Figure 13:**
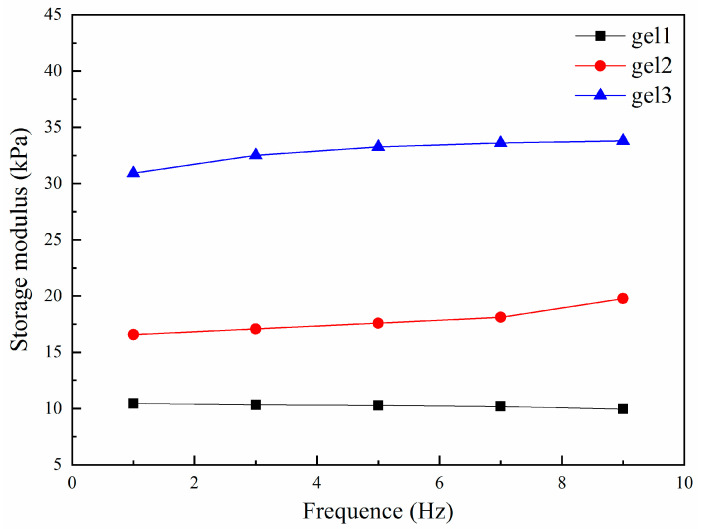
Energy storage modulus curve of gels.

The energy storage modulus of hydrogels was investigated to respond to the physico-mechanical properties of the gels, and the three gels were fully swollen and equilibrated in the secondary deionized water at 37 °C before the measurement. The curves of the energy storage modulus of the gels with different crosslinking modes with respect to the frequency were measured by the DMA (dynamic mechanical analysis), and the results are shown in Figure 13 and Table 4. It can be seen that the energy storage modulus curves of the three gels change insignificantly with increasing oscillation frequency, at a frequency of 9 Hz. The energy storage moduli of gel1, gel2, gel3 were 9.96 kPa, 19.77 kPa, and 33.81 kPa, respectively. Gel1, gel2, and gel3 show a gradual increase in the energy storage modulus, and the energy storage modulus of the triple-crosslinked hydrogel is significantly better than that of the other two gels, so gel3 has the best mechanical strength. The mechanical strength of the gel is opposite to the swelling rate, which is consistent with the results of a comparison regarding the swelling rate of the gel measured previously.

In addition, the loss angle tangent of the gel was also determined, which indicates the viscoelasticity of the material. The larger the loss angle tangent, the greater the viscosity of the material; the smaller the loss angle tangent, the greater the elasticity of the material. Gel1, gel2, and gel3 at a frequency of 9 Hz had a loss angle tangent value of 0.1000, 0.0198, and 0.0338, respectively. The loss angle tangent is very small, indicating that the gel in the action of the external force to the elasticity of the main has better toughness.

### 2.10. Sustained Drug Release of the Gels

**Figure 14:**
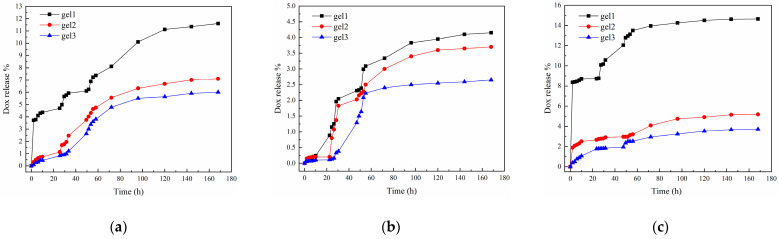
(**a**) Drug release rates of the gels at pH = 5.3, 37 °C; (**b**) drug release rates of the gels at pH = 7.4, 37 °C; (**c**) drug release rates of the gels at pH = 7.4, 40 °C.

The literature suggests that many malignant tumors are surrounded by tissue environments that are either hyperthermic (*T* of about 40 °C) or weakly acidic (pH of about 5.3) [78,79], so we investigated the effect of PBS buffer on the slow release of the drug from the gel by simulating the human tissue environment and using PBS buffer as a solution by changing the pH and temperature, respectively, We set the following conditions: (1) pH = 5.3, *T* = 37 °C; (2) pH = 7.4, *T* = 37 °C; (3) pH = 7.4, *T* = 40 °C. This was to determine the delays in drug release from the gel, which were obtained, as shown in Figure 14a–c and Table 5, where (b) shows the simulated normal physiological parameters of the human body; (a) the effect of varying pH on drug release was studied; (c) the effect of varying temperature on drug release was studied. As can be seen in Table 5, the highest drug release in all conditions is gel1 of (c), which is 14.65%, and it can be seen that the drug-carrying gel has a slow drug release, which is due to the fact that doxorubicin hydrochloride is a hydrophobic drug [80] that mainly aggregates in the hydrophobic chain region and does not diffuse well in water. Under physiological parameter conditions (b), the cumulative drug release of gel1, -2, and -3 after 168 h was 4.15%, 3.70%, and 2.65%, respectively, which were less than 5%, indicating that the drug-carrying gel had less drug leakage in normal tissues, which reduced the toxicity to normal tissues. When the environment was changed to be slightly acidic (a), the cumulative drug release of gel1, -2, and -3 after 168 h was 11.60%, 7.10%, and 6.00%, respectively. The increase in drug release was 7.45%, 3.4%, and 3.35%, respectively, compared to (b). There was also a decrease; when the ambient temperature (c) was varied, the cumulative drug release after 168 h was 14.65%, 5.18%, and 3.71% for gel1, -2, and -3, respectively; the increase in drug release was 10.5%, 1.48%, and 1.06%, respectively; and there was also a gradual decrease. The drug release decreased with the increase in crosslinking mode, and this result is also consistent with the results of temperature-sensitive and pH-sensitive swelling tests of the gels. Through the study of the drug’s slow release in the gel, it was found that the two parameters of temperature and pH in the human environment have an effect on drug slow release. According to Table 5, it can be observed that pH variation has a greater impact on drug release from the gel compared to temperature variation. Specifically, the gel releases more drug in a mildly acidic environment than in a high-temperature environment. This indicates that in a slightly acidic environment, the drug is able to act faster on the cancer cells, and the targeting property is manifested more greatly. Through the study, it was shown that the triple-crosslinked hydrogel did not have much effect on the controlled release of hydrophobic drugs in the case of improving the mechanical properties and reducing the swelling capacity, and the cumulative amount of the drug released after 168 h was less than 1% compared with the double-crosslinked hydrogel, which is of great significance in gel drug delivery. Our future research will focus on enhancing the drug release rate of the trimethyl crosslinked hydrogel to achieve a faster and more efficient targeting of cancer cells.

## 3. Conclusions

In this study, three types of hydrogels were synthesized according to the crosslinking method, namely physically crosslinked hydrogels, physico-chemical double-crosslinked hydrogels, and physico-chemical click triple-crosslinked hydrogels. The temperature-sensitive monomers MEO_2_MA and OEGMA, and the pH-sensitive monomer DEAEMA, were polymerized into the gels by free-radical polymerization of the three hydrogels, and the swelling tests of the three hydrogels indicated that the hydrogels had good temperature sensitivity, pH sensitivity, reversibility, and amphiphilicity. The thermal analysis and mechanical properties of the gels showed that the thermal stability and mechanical strength of the gels increased with an increase in the crosslinking mode under the same degree of PLA polymerization, realizing the purpose of the triple-crosslinked hydrogels to achieve a high melting point and better mechanical properties; the analysis of the drug-carrying properties of the gels indicated that the triple-crosslinked hydrogels have good biocompatibility, high thermal stability, and good mechanical properties, in addition to the controlled release of hydrophobic drugs, which has a wide range of prospects for applications in the field of biomedicine.

## 4. Materials and Methods

### 4.1. Materials

D-lactide (D-LA, 98%) was purchased from Aladdin Chemicals Co., Ltd. (Shanghai, China). Hydroxyethyl methacrylate (HEMA, 99%), 1,8-diazabicyclic [5.4.0] undeca-7-ene (DBU, 99%) and 2-bromoisobutanoyl bromide (BIBB, 98%) were purchased from Beijing Balinway Technology Co., Ltd. (Beijing, China). L-lactide (L-LA, 98%), 2-methyl-2-acylate-2-(2-methoxy-ethoxy) ethyl ester (MEO_2_MA, 97%), oligoethylene glycol methyl ether methacrylate (OEGMA, 95%), azodiisobutyronitrile (AIBN, 98%) and propargyl bromide(C_3_H_3_Br, 98%) were purchased from Shanghai Maclin Biochemical Technology Co., Ltd. (Shanghai, China). Triethylamine (TEA, 99%), sodium azide (analytical pure) and sodium hydride (analytical pure) were purchased from Shanghai Sinophosphoric Chemical Reagent Co., LTD. (Shanghai, China). *N*,*N*’-dimethylformamide (DMF) ultra-dry solvent and tetrahydrofuran (THF) ultra-dry solvent were purchased from Anaeji Chemical Co., Ltd. (Shanghai, China). Dichloromethane (DCM) was purified, and the experimental water was secondary deionized water.

### 4.2. Synthesis

#### 4.2.1. Synthesis of the Steric Complex HEMA-(PLLA-PDLA)_n_

Under a dry nitrogen atmosphere, hydroxyethyl methacrylate (HEMA) (28 μL, 0.25 mmol), D-lactide (0.5 g, 3.45 mmol), and dry DCM (30 mL) were added to a 100 mL dry, three-necked round-bottomed flask. The mixture was stirred until all monomers were dissolved, then DBU (50 μL, 0.33 mmol) was added. After stirring at room temperature overnight, the reaction was terminated by addition of excess benzoic acid under ice bath conditions. The reaction mixture was precipitated twice in hexane and finally dried to remove the solvent to obtain the macromonomers HEMA-PLLA_30_. HEMA-PDLA_30_ was synthesized in the same way as HEMA-PLLA_30_. The prepared macromonomers HEMA-PLLA_30_ and HEMA-PDLA_30_ were each 0.1 g, put into a dry 10 mL round-bottomed flask, and 3 mL THF was added to dissolve them; then, they were ultrasonicated for 120 min to ensure that intermolecular hydrogen bonding was formed between PLLA and PDLA, and the steric complexation was complete. Then, the solvent was removed to obtain the stereocomplex HEMA-(PLLA-PDLA).

#### 4.2.2. Synthesis of the Azide Polymer HEMA-(PLLA-PDLA)-N_3_

The stereocomplex HEMA-(PLLA-PDLA)_n_ 0.2 g was placed into a flask, 8 mL of DMF solvent was added to dissolve it, and then triethylamine was added, and the reaction was carried out under the protection of nitrogen for 30 min. Then, the flask was put into an ice bath, and 2-bromoisobutyryl bromide was added drop by drop to 0.2 mL; the reaction was carried out under the protection of nitrogen for 24 h. At the end of the reaction, sodium azide 0.3 g was added, and the reaction was carried out at room temperature for 72 h. The product was then purified by transferring it into a dialysis bag with a molecular weight cut-off of 500 g/mol for 3 days, and the product HEMA-(PLLA-PDLA)-N_3_ was obtained as a white powder after freeze-drying.

#### 4.2.3. Synthesis of the Alkynylated Polymers HEMA-(PLLA-PDLA)-Alkyne

The stereocomplex HEMA-(PLLA-PDLA)_n_ 0.2 g was placed into a flask and 8 mL of THF was added to dissolve it, followed by the addition of sodium hydride (28 mg) after stirring, until no bubbles were produced. Propargyl bromide (240 μL) was added, and the reaction was carried out at room temperature for 48 h. The product was then transferred into a dialysis bag, with a molecular weight cut-off of 500 g/mol, and dialyzed for 3 days. The light-yellow powdery product HEMA-(PLLA-PDLA)-alkyne was obtained after freeze-drying.

#### 4.2.4. Synthesis of Physically Crosslinked Hydrogels

We placed the stereocomplex HEMA-(PLLA-PDLA)_n_ 0.2 g into a flask with the temperature-sensitive monomer MEO_2_MA, OEGMA, and pH-sensitive monomer DEAEMA into the system proportionally, with ultrasonication for 30 min to make the stereocomplexed macromolecule monomers completely dissolved. We then added the catalyzer AIBN (0.0020 g), the reaction system was hermetically sealed, and a vacuum pump was used to pump the gas to create an oxygen-free environment. Then, nitrogen was passed in to further remove the oxygen from the reaction system, and this was repeated three times with pumping–vacuuming–nitrogen pumping. Finally, the flask was placed into an oil bath at 65 °C for 3 h to form a hydrogel.

#### 4.2.5. Synthesis of Physico-Chemical Double-Crosslinked Hydrogels

The stereocomplex HEMA-(PLLA-PDLA)_n_ 0.2 g was placed into a flask, and the temperature-sensitive monomers MEO_2_MA, OEGMA, the pH-sensitive monomer DEAEMA, and the chemical crosslinking agent EGDMA were added proportionally to the system. The next steps were consistent with the steps of synthesizing the complexed hydrogels and will not be repeated.

#### 4.2.6. Synthesis of Physicochemical Click Triple-Crosslinked Hydrogels

HEMA-(PLLA-PDLA)-N_3_ and HEMA-(PLLA-PDLA)-alkyne was put into a round-bottomed flask according to a certain ratio, with DMF as the solvent, and a vacuum pump was utilized to pump gas to create an anaerobic environment. Nitrogen was then passed in to further remove the oxygen in the reaction system, and this pumping–nitrogen pumping was repeated three times, after which CuBr was rapidly added and clicked. After the reaction was complete, it was transferred into a dialysis bag with a molecular weight cut-off of 500 g/mol for 3 days and freeze-dried, and 0.2 g of the product was placed in a round-bottomed flask; the temperature-sensitive monomers MEO_2_MA, OEGMA, the pH-sensitive monomer DEAEMA, and the chemical crosslinking agent EGDMA were added to the system proportionally, and then the catalyzer AIBN was added (0.0020 g), the system was sealed, and the hydrogel was obtained after complete reaction at 65 °C in an oil bath under nitrogen atmosphere.

### 4.3. Structural Characterizations

#### 4.3.1. Nuclear Magnetic Resonance Hydrogen Spectrum (^1^H NMR)

Measurements were made using a 400 MHz (JEOL) nuclear magnetic resonance spectrometer, specification JNM-ECZ400S/L1, manufactured by Japan Electronics Corporation, with the solvent CDCl_3_.

#### 4.3.2. Infrared Spectrum (FT-IR)

Measured by a near-infrared–mid-infrared spectrometer with a wave number of 12,000 cm^−1^–400 cm^−1^ produced by Bruker, Germany, the samples were fully ground and dried with KBr before measurement and then pressed at room temperature for measurement.

#### 4.3.3. X-ray Diffraction Curves (XRD)

Powder X-ray diffractometer with specifications of Bruker D8 Advance from Bruker, Germany, was used for the determination, and the samples were freeze-dried.

### 4.4. Character Determination

#### 4.4.1. Swelling Test of Gels

The temperature sensitivity and pH sensitivity of the gels can be shown by the swelling and deswelling rates of the gels at different temperature and pH conditions. The prepared gel was freeze-dried and cut into pieces, weighed, immersed in (1) *T* = 25 °C, pH = 7, (2) *T* = 37 °C, pH = 7, (3) pH = 5, *T* = 37 °C, and (4) pH = 9, *T* = 37 °C of secondary deionized water, taken out at fixed intervals, and the filter paper sucked out the water on the surface of the gel. This was weighed and measured, and the swelling and deswelling rates were calculated according to the following formulas:

*Swelling ratio* = (*W_t_* − *W_d_)*/*W_d_* (1)

where *W_t_* is the mass (g) of the gel at the time of swelling to t, and *W*_d_ is the mass (g) of the dried gel.

*Deswelling ratio* = (*W_t_* − *W_d_*)/*W_s_* (2)

where *W_t_* is the gel mass (g) at the time of swelling to *t*, *W*_d_ is the gel mass (g) after drying, and *W_s_* is the gel mass (g) after complete swelling.

#### 4.4.2. Reversibility Testing of Gels

The reversibility of the gel can be illustrated by the rate of swelling and deswelling. The gel was freeze-dried, cut into pieces, and placed in secondary deionized water with a pH = 7. The temperature was constantly switched between 25 °C and 40 °C for a fixed period of time, and the swelling and deswelling rates were calculated using Formulas (1) and (2).

#### 4.4.3. Amphiphilicity Testing of Gels

The amphiphilicity of the gel can be expressed by the gel’s swelling rate. The gel was freeze-dried and cut into pieces, put into secondary deionized water and tetrahydrofuran at pH = 7, removed at fixed intervals, and filter paper absorbed the moisture on the surface of the gel. We then weighed its mass, and the swelling rate was calculated according to Formula (1).

#### 4.4.4. Thermal Stability Testing of Gels

The thermal properties of the gels were tested using a thermal analyzer specification STA449F5 manufactured by NETZSCH Instruments GmbH, Germany. About 5 mg of dried gel sample was weighed in the crucible of the instrument and scanned in a range of 25–600 °C under nitrogen atmosphere, purging at a heating rate of 10 °C/min.

#### 4.4.5. Scanning Electron Microscopy (SEM) Analysis of Gels

The morphology of the gel was analyzed using a TM3000 benchtop scanning electron microscope manufactured by Hitachi High-Technologies Naka Corporation, Japan. Before the test, the gel was completely swollen in secondary deionized water to equilibrium, rapidly cooled with liquid nitrogen to maintain its morphology, freeze-dried, and then placed into the instrument to observe the morphology.

#### 4.4.6. Mechanical Properties Testing of Gels

Dynamic viscoelastic spectrometer (DMA) with specification Q850 manufactured by TA Instruments, New Castle, United Kingdom, was used for testing. The gels were allowed to swell completely to equilibrium in secondary deionized water at 37 °C prior to testing, and cylindrical samples were made with a perforator and tested using a compression mold under an oscillating frequency scan at 37 °C, an amplitude of 10 μm, and an initial pressure of 0.01 N. The gels were then subjected to an oscillating frequency scan at 37 °C.

#### 4.4.7. Drug-Carrying Capacity Testing of Gels

The drug-carrying properties of the gels can be obtained by UV analysis.

Preparation of drug-carrying gel: 5 mg of dried gel was weighed and completely immersed in 1 mg/mL doxorubicin hydrochloride solution for 72 h at room temperature, removed and cleaned with THF to remove the drug residue on the surface of the gel, placed at room temperature for 24 h, and then put into a vacuum drying oven at 40 °C for 4 days for use.

Determination of maximum absorption wavelength: 50 μg/mL of doxorubicin hydrochloride solution was prepared in PBS buffer at pH = 7.4 as a blank control group, and its maximum absorption wavelength was obtained as 233 nm by spectral scanning between 200 and 600 nm with a UV-visible spectrophotometer model UV-1901.

Determination of standard curve: Doxorubicin hydrochloride solutions in the range of 1–50 μg/mL were prepared using PBS buffer at pH = 7.4 as solvent. The absorbance of each solution was measured at a maximum absorption wavelength of 233 nm, and the standard curve and linear regression equation were obtained after fitting the curve.

*Abs* = 0.0560*c* + 0.0046, *R*^2^ = 0.9975 (3)

where *Abs* is the absorbance of doxorubicin hydrochloride at a wavelength of 233 nm, and *c* is the concentration of doxorubicin hydrochloride.

Determination of cumulative drug release: The drug-carrying gel was immersed in (1) pH = 7.4, *T* = 37 °C, (2) pH = 5.2, *T* = 37 °C, (3) pH = 7.4, *T* = 40 °C, in a flask containing 150 mL of PBS buffer and then put into a shaker to remove 3 mL of buffer at intervals to measure the absorbance at maximum absorbance. The flask was then replenished with an equal amount of the corresponding fresh buffer, and the corresponding buffer concentration was derived from the measured standard curve; the cumulative release of doxorubicin hydrochloride was then calculated according to the formula:

(4) $$Cumulative\;release\;\left(\%\right)=\frac{V_{e}\sum_{1}^{n-1}C_{i}+{V_{0}C}_{n}}{m_{drug}}\times 100$$

where $V_{e}$ is the volume of solution obtained from the PBS buffer solution each time, that is, 3 mL; $V_{0}$ is the total volume of the released solution, 150 mL; *C_i_* is the concentration of the gel released when the solution is removed for the first time; *m*_drug_ is the total mass of doxorubicin hydrochloride loaded into the gel, 5 mg.

## Figures and Tables

**Table 1:** Synthesis data of gels.

Samples	HEMA-(PLLA-PDLA) (mmol)	EGDMA (%)	HEMA-(PLLA-PDLA)-Alkyne (w/g)	HEMA-(PLLA-PDLA)-N_3_ (w/g)	Crosslinking Types
gel1	0.2	0	0	0	Physical crosslinking
gel2	0.2	0.6	0	0	Physical-chemical dual crosslinking
gel3	0	0.6	0.2	0.16	Physical-chemical-clicking triple crosslinking

The molar ratio of MEO_2_MA to OEGMA is 9:1; the molar ratio between DEAEMA and P(MEO_2_MA-*co*-OEGMA) is 3:7.

**Table 2:** Swelling rate for gels at 25 °C and pH = 7.

	Time (h)	0	0.5	1	1.5	2	3	4	5	6	7	8
Samples												
gel1		0	16.94	31.07	39.88	47.39	57	61.1	63.12	64.42	61.4	60.94
gel2		0	4.99	5.38	5.42	5.41	5.56	5.53	5.56	5.59	5.4	5
gel3		0	1.99	2.65	2.98	3.15	3.29	3.32	3.32	3.18	3.16	3.14

Swelling rate in %.

**Table 3:** Swelling rate for gels at 37 °C and pH = 5.

	Time (h)	0	0.5	1	1.5	2	2.5	3	4	5	6	7	8
Samples													
gel1		0	14.06	26.44	40.52	46.56	51.07	54.81	56.89	60.54	61.59	62.23	63.14
gel2		0	1.82	2.37	2.69	2.91	3.06	3.15	3.34	3.47	3.54	3.66	3.71
gel3		0	0.48	0.56	0.65	0.71	0.81	0.85	0.96	1.07	1.16	1.09	1.20

Swelling rate in %.

**Table 4:** Storage modulus of gels.

	Frequence (Hz)	1	3	5	7	9
Samples						
gel1		10.44	10.33	10.28	10.19	9.96
gel2		16.57	17.07	17.58	18.11	19.77
gel3		30.92	32.52	33.26	33.61	33.81

Storage modulus in kPa.

**Table 5:** Drug release rate from gels under different conditions at 168 h.

	Reaction Conditions	pH = 5.3, 37 °C	pH = 7.4, 37 °C	pH = 7.4, 40 °C
Samples				
gel1		11.60	4.15	14.65
gel2		7.10	3.70	5.18
gel3		6.00	2.65	3.71

Drug release rate in %.

## Data Availability

The data presented in this study are openly available in article.
